# An Adaptive Augmented Vision-Based Ellipsoidal SLAM for Indoor Environments

**DOI:** 10.3390/s19122795

**Published:** 2019-06-21

**Authors:** Elfituri S. Lahemer, Ahmad Rad

**Affiliations:** Autonomous and Intelligent Systems Laboratory, School of Mechatronic Systems Engineering, Simon Fraser University, Surrey, BC V3T 0A3, Canada; arad@sfu.ca

**Keywords:** EKF/Ellipsoidal SLAM, robotic augmented reality, Nao Humanoid robot, adaptive augmented Ellipsoidal- SLAM, landmark

## Abstract

In this paper, the problem of Simultaneous Localization And Mapping (SLAM) is addressed via a novel augmented landmark vision-based ellipsoidal SLAM. The algorithm is implemented on a NAO humanoid robot and is tested in an indoor environment. The main feature of the system is the implementation of SLAM with a monocular vision system. Distinguished landmarks referred to as NAOmarks are employed to localize the robot via its monocular vision system. We henceforth introduce the notion of robotic augmented reality (RAR) and present a monocular Extended Kalman Filter (EKF)/ellipsoidal SLAM in order to improve the performance and alleviate the computational effort, to provide landmark identification, and to simplify the data association problem. The proposed SLAM algorithm is implemented in real-time to further calibrate the ellipsoidal SLAM parameters, noise bounding, and to improve its overall accuracy. The augmented EKF/ellipsoidal SLAM algorithms are compared with the regular EKF/ellipsoidal SLAM methods and the merits of each algorithm is also discussed in the paper. The real-time experimental and simulation studies suggest that the adaptive augmented ellipsoidal SLAM is more accurate than the conventional EKF/ellipsoidal SLAMs.

## 1. Introduction

Navigation in structured and unstructured environments is a central activity in many robotics applications. The simultaneous localization and mapping (SLAM) [1] problem has been recurrently studied by researchers around the world in the last three decades. The problem is essentially creating (updating) a map of an unknown environment along with the robot poses concurrently. The question can be poised for static as well as dynamic settings, indoor or outdoor, for different robotic platforms, and for different environments such as land, underwater, or the sky [2]. Although, for every robotics platform, the type of sensors and the environment itself affect which mapping and navigation approach fits best, resolving uncertainty in the robot and landmark positions have been on-going research questions. This uncertainty has long been one of the prime bottlenecks towards building robust systems in all domains to accomplish the task of autonomous navigation. As such, a viable solution is regarded as a prerequisite for efficient implementation of a robotic solution to a particular application domain. Consequently, in recent years, a great effort has been devoted to the development of efficient solutions to the SLAM problem [3].

By adopting a landmark-based description of the environment, autonomous navigation including mapping and localization problem can be cast as a state estimation problem for an uncertain dynamic system based on noisy measurements. Depending on the assumptions of the uncertainty, the estimation problem can be tackled in different ways. When a statistical description of the noise is adopted for mapping and localization processes, the uncertainty is handled by a relevant probabilistic technique which is arguably the dominant paradigm for robotics navigation and SLAM. These approaches represent uncertainty and ambiguity through explicit “acquired beliefs” using probability theories and form robust control choices relative to the remaining uncertainty in the model. Standard solutions are provided by the extended Kalman filter (EKF) [4,5,6] or other probabilistic techniques [7,8]. All the methods based on the EKF generally model the uncertainty in robot dynamics and the measurement process as zero-mean, white Gaussian noise. Unfortunately, as the robot moves away into increasingly unstructured and unknown environments, such assumptions may not hold and the ability to deal with uncertainty becomes a crucial component for designing successful SLAM systems. Moreover, even when these assumptions are fulfilled, the EKF may not converge. This assumption is no longer valid when the real error distributions are not Gaussian. These considerations have motivated researchers to adopt alternative techniques. One of the most popular is the set-theoretic or set-membership estimators, whose main feature is that the estimation uncertainty is represented by bounded sets in the state space [9,10]. Its basic premise is to assume hard bounds on the noise, and consequently hard bounds on the state estimate process. In contrast to the KF or EKF, noise sources are assumed to be stochastic, and the highest probability state estimate and covariance are recursively calculated. The exact shape of this set is, in general, very complicated and hard to obtain. Therefore, it is usually approximated by simpler geometric shapes, such as box, ball, ellipsoid, orthotope, and zonotopes. Among them, the ellipsoidal estimation seems to be more popular because of its analogy to the covariance in the stochastic methods [11,12]. The general set membership approach is an appropriate choice to solve the state estimation problem with bounded disturbances [13]. The set membership estimation method recursively computes an output set in which no point is more likely to be the actual state than any other point, but does guarantee that the actual state lies within this set [9]. The resulting algorithm has a prediction-correction structure in-time update and observation update, which is similar to a Kalman filter. This approach was initially introduced by Schweppe and Witsenhausen [14]. These authors proposed solutions to set estimation problems of linear systems with specific hypotheses on the boundaries. Under the assumption of unknown but bounded (UBB), the estimation process was carried out in terms of feasible sets i.e.; sets containing the robot pose and the landmark positions. As such, these estimation algorithms are able to provide guaranteed set-valued estimates of the robot configuration as well as of the landmark positions. Ellipsoid methods require less information to represent the uncertainty as compared to polytope methods, and are perhaps more intuitive because of their analogy to the covariance in stochastic estimation.

The approach is to cast the nonlinear dynamics in a way that is suitable for implementation within the linear set membership filter framework. Specifically, the nonlinear dynamics are linearized about the current estimate in a manner that is similar to the EKF [11]. The remaining terms are then bounded using interval mathematics, and they are incorporated into the algorithm as additions to the process or sensor noise bounds. This allows the solution to be guaranteed for nonlinear systems so long as a bound on the nonlinear term is guaranteed. The method is compatible with many current robust control methods that require hard bounds, as well as with planning algorithms that require guaranteed uncertainty information. It is also computationally efficient for online recursive implementation. The new algorithm is termed the extended set membership filter (ESMF). 

Despite the benefits of the ellipsoidal SLAM algorithm in terms of relaxing the noise in a dynamic system and measurements and the convergence as an estimator and no explicit assumption for Gaussian’s structure, it is subjected to its own concerns. The main issue is how to calibrate the ellipsoidal SLAM parameters and how to improve its accuracy. The data association problem and computational cost problem are also other issues to be considered. Another issue is that in SLAM, the state vector contains only the landmark position and robot poses and SLAM alone is not expected to convey more information about landmarks details. The map representation does not include any information about landmark description in the environment and this can lead to false positives in the data association.

In this paper, we introduce the notion of the robotic augmented reality (RAR) technique to improve the performance of the EKF/ellipsoidal SLAMs and to overcome the above issues. Adaptive augmented EKF/ellipsoidal SLAMs have been implemented and tested in real-time. SLAM implemented on a NAO humanoid robot. Those who have worked with this particular humanoid robot are aware that it is generally not easy to interface external sensors; our goal, hence, is to design a SLAM system on NAO using its own camera. 

The outline of the paper is organized as follows: in Section 2, we review the related works and highlight the current state-of-art in SLAM and its main challenges. In Section 3, we present the detailed architecture of the proposed system. We will then include simulation studies and discuss the merits of the proposed architecture in Section 4. We conclude the paper in Section 5.

## 2. Related Studies

Readers not familiar with the origin, history, and derivation of SLAM may refer to classical and recent papers on SLAM and its development [15,16,17]. The extended Kalman filter-based SLAM, which has been also implemented in this work, is the most popular implementation [5,6,18]. The importance of this approach as compared to the Kalman filter remains in its ability to represent non-linear models. This capability is essential, as almost all navigation problems can be modeled as non-linear problems. However, highly non-linear problems tend to have erroneous approximations and may cause the failure of an EKF-based SLAM. This led to the idea of using an unscented Kalman filter (UKF)-based SLAM, by Julier and Uhlmann [19]. The UKF would perform better linearization and therefore improve map building by reducing errors. [20] Implementing the adaptive UKF solution will significantly reduce the error and improve the accuracy of the navigation system. This was followed by FastSLAM [21], which uses particle filter-based techniques to perform map building. Errors due to non-linearity and high computational cost led to a host of sub-map-based SLAM algorithms that divide the map into local sub-maps which perform partial updates. Recently, research has shifted towards solving the SLAM problem with sparse optimization techniques which provide a higher efficiency SLAM yet do not suffer from errors. In the study [22], the SLAM is solved via two parallel tasks: pose estimation, and map optimization. Both tasks concurrently operate as a service in the Cloud. It is clear that all probabilistic feature-based online/full SLAM solutions include the Kalman filter and its variants such as the information filter, particle filter, and graph optimization solutions that all assume a Gaussian distribution of uncertainties. 

To cope with non-white, non-Gaussian noise, a set-theoretic approach to the problem of tracking a mobile robot, based on angular measurements, has been introduced in [23] under the assumption of bounded errors to obtain information fusion via set intersections. This approach has been later extended to a mixed stochastic/set-membership framework [24], in which a probabilistic uncertainty description is associated to ellipsoidal approximations of the admissible robot poses.

As far as the SLAM problem is concerned, preliminary ideas on how to deal with bounded errors are given in [25,26,27]. These papers present set-theoretic approaches to localization only, which has been recently developed and successfully applied to several problems in mobile robotics platforms.

Garulli and Vicino [28] present a simple localization way using a polytope set. The polytope method been applied for SLAMs in [29,30,31]. They used the interval analysis and polytope set memberships to find robot trajectory. The algorithms suffer from large computational costs and the correlations between the robot and landmark positions are lost due to decomposition simplification [5]. In fact, the polytope method increases the inequality number over time. It is not suitable for online application.

The ellipsoid method for state estimation with bounded noise was first introduced by Schweppe [11], Fogel and Huang [14]. Many simulated technical papers followed this paper. Weyer and Campi [32] obtained confidence ellipsoids which were valid for a finite number of data points. Ros et al. [33] presented an ellipsoid propagation such that the new ellipsoid satisfies an affine relation with another ellipsoid. 

Intervals of real numbers are used to represent the uncertainty in [30,31]. It translates full SLAM problem in terms of a constraint satisfaction problem. It uses interval analysis and contraction techniques to find the minimal envelope of robot trajectory and the minimal sets to enclose the landmarks but the association between landmarks and observations is not solved. In [30], the algorithms only works off-line due to the large computational cost of contraction. The adaptive augmented ellipsoidal SLAM and RAR simplifies and solved the data association problem and reduce the computational cost. Also, the implemented adaptive augmented ellipsoidal SLAM is able to work and calibrate its parameters online. Our implemented adaptive augmented ellipsoidal SLAM can work with limited sensor requirements.

## 3. Methodology and Implementation 

### 3.1. SLAM Solution with Extended Kalman Filter (EKF-SLAM)

Autonomous navigation is among the main areas of research in the mobile robotics field which often requires a SLAM solution. SLAM algorithms allow a robot to map its environment while concurrently localizing itself within that environment. The trajectory of the robot and the position of the landmark in the map are estimated without the need for a priori knowledge. 

**Figure 1:**
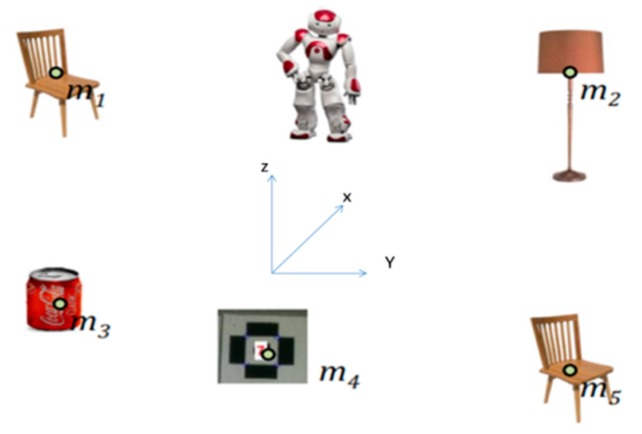
NAO robot in indoor environment performing SLAM.

By considering a landmark-based description of the environment, the SLAM can be considered a state estimation problem for an uncertain dynamic system, based on noisy measurements. Consider a robot moving in an unknown environment (NAO robot), observing a number of landmarks through its embedded sensor (Figure 1). 

The robot requires a control signal $u_{t}$ {$u_{1}$, $u_{2}$, …, $u_{k}$} to be able to move from position $x_{t-1}$ to $x_{t}$ using the IMU (inertial measurement unit) which has some uncertainty in its measurement, and the robot takes some observation $z_{t}$ using its external sensors, e.g., lasers. The robot pose $x_{t}$ and/or the landmark positions $m_{i}$ are concurrently estimated. The goal in every iteration is to find the posterior data which refers to the estimated data of robot position (or the whole trajectory) and all landmarks positions together and reduce the measurement errors and observation error. It can be represented in one vector the environment map containing a list of objects.

EKF-SLAM was the first algorithm developed by Smith to solve SLAM problem and is still one of the most influential and widely used SLAMs [34]. It concurrently solves the online SLAM problem where the map is feature-based. The EKF essentially linearizes the non-linear functions around the current state which is accomplished by the Taylor expansion technique. After this linearization process, a classical Kalman filter is employed to estimate states. The EKF-SLAM estimates the state from a series of noisy measurements (movements and observations), where the noise distribution is assumed Gaussian. The probability density function (PDF) of the estimated state is Gaussian, as well:

(1) $$p\left[x_{t},m|z_{0:t},u_{0:t}\right]=N\left(\left\{x_{t},m_{i}\right\},\mu_{t,}\Sigma_{t}\right)=N\left(s_{t},\mu_{t,}\Sigma_{t}\right),$$

where N is a Gaussian probability density with its mean $\mu_{t}$ and covariance matrix $\Sigma_{t}$. Therefore, for every iteration of the EKF filter, the uncertainty of the state $s_{t}$ will be represented through a column vector $\mu_{t}$ of size n and a covariance matrix $\Sigma_{t}$ of size n × n, where n is the dimension of the state. In the EKF-SLAM, the probability density of the state transition $p\left[s_{t}|s_{t-1},u_{t}\right]$ must be Gaussian, this means that given the state $s_{t-1}$ at time t − 1 and the movement $u_{t}$ at time t, the transition of the state can be written as [35]:

(2) $$s_{t}=f\left(u_{t},s_{t-1}\right)+v_{t}$$

where $v_{t}$ is an additive Gaussian noise with zero mean and covariance matrix $Q_{t}$ that models the uncertain in the state transition, i.e., P($v_{t}$) ∼ N (0, $Q_{t}$). Since the motion model depends only on the previous robot pose and on the current movement $u_{t}$, we can omit in the equation the map vector, i.e.:

(3) $$x_{t}=f\left(u_{t},x_{t-1}\right)+v_{t}$$

The function f doesn’t modify the map. The probability density function of the observation $z_{t}$ must be Gaussian as well, that is:

(4) $${\hat{z}}_{t}=h\left(s_{t},m_{i}\right)+w_{t}$$

where h is a function that maps the current state in an expected observation ${\hat{z}}_{t}$ given the landmark $m_{i}$ associated to ${\hat{z}}_{t}$, $w_{t}$ is an additive Gaussian noise with zero mean and covariance matrix $R_{t}$ that models the observation noise, i.e., P($v_{t}$) ∼ N (0, $R_{t}$).

In the 2D case, the state $s_{t}$ at time t can be represented by the following column vector:

(5) $$s_{t}={\left(p_{x,t,}\;p_{y,t,}\;p_{\theta ,t,}\;m_{x,1,}\;m_{y,1,}\;m_{x,2,}\;m_{y,2,}\;\ldots \;\ldots \;\ldots \;m_{x,N,}\;m_{x,N,}\;m_{s,N,}\right)}^{T}$$

where $x_{t}$ = $\left(p_{x,t,}\;p_{y,t,}\;p_{\theta ,t,}\right)$ denotes the robot’s coordinate, $m_{x,i,}m_{y,i,}$ are the coordinates of the i −th landmark $m_{i}$ with $m_{s,i}$ its distinctive signature, i = 1, …, N. 

The state vector $s_{t}$ contains only landmark positions ($m_{x,i,}m_{y,i,}$) and the robot pose $x_{t}.$ SLAM is not expected to convey more information about the landmark’s description in the environment. 

In the extended Kalman filter, the f() *and* h() functions are linearized using first order Taylor expansion around the state of the system in order to apply the Kalman equations. In the case of the state transition function f we can write:

(6) $$f\left(u_{t},s_{t-1}\right)≃f\left(u_{t},\mu_{t-1}\right)+F_{t}\left(s_{t}-\mu_{t-1}\right)$$

where $F_{t}$ is the n × n jacobian matrix of the function f (n is the dimension of the state). The jacobian usually depends on $u_{t}$ and $\mu_{t-1}$:

(7) $$F_{t}=\nabla_{s_{t-1}}\;f\left(u_{t},s_{t-1}\right){|}_{s_{t-1}=\mu_{t-1,s_{t}=\mu_{t}}}$$

The Kalman filter is completed in two steps: prediction and correction [36,37]. In the SLAM problem, during the prediction step the state at time t is updated according to the motion model:

(8) $$\bar{\mu_{t}}=f\left(u_{t},\mu_{i-1}\right)$$

(9) $$\bar{\mu_{t}}=F_{t}\Sigma_{t-1}\;F_{t}^{T}+Q_{t}$$

where $\mu_{i-1}$ and $\Sigma_{t-1}$ are the mean and covariance of the state at time t−1, respectively. During the correction step, for every observation $z_{t}$ associated with the landmark $m_{i}$, it is computed an expected observation ${\hat{\;z}}_{t}=h\left(\bar{\mu_{t}},m_{i}\right)$ and a corresponding Kalman gain (11) that specifies the degree to which the incoming observation corrects the current state estimation (Equations (12) and (13))

(10) $$S_{t}=H_{t}\Sigma_{t}^{-}\;H_{t}^{T}+R_{t}$$

(11) $$S_{t}=H_{t}\Sigma_{t}^{-}\;H_{t}^{T}+R_{t}$$

(12) $$\mu_{t}=\bar{\mu_{t}}+K_{t}\left(z_{t}-h\left(\bar{\mu_{t}},m_{i}\right)\right)$$

(13) $$\Sigma_{t}=(1-K_{t}H_{t})\Sigma_{t}^{-}$$

where $H_{t}$ is the jacobian of the function h:

(14) $$H_{t}=\nabla_{s_{t}}\;h\left(u_{t},m_{i}\right){|}_{s_{t-1}=\bar{\mu_{t}}},m_{i}=m_{i}$$

The standard formulation of the EKF-SLAM solution is not robust to incorrect association of observations to landmarks: an accurate data association is then desirable [38]. 

In this work, the noise in motion model is modeled by white Gaussian noise with zero mean and covariance value N (0, $Q_{t}$) = diag [0.015 0.015 0.0001] and for observation model is N (0, $R_{t}$)= diag [0.008 0.001].

### 3.2. Ellipsoidal Set Membership Filter Method for SLAM (Ellipsoidal SLAM) 

**Figure 2:**
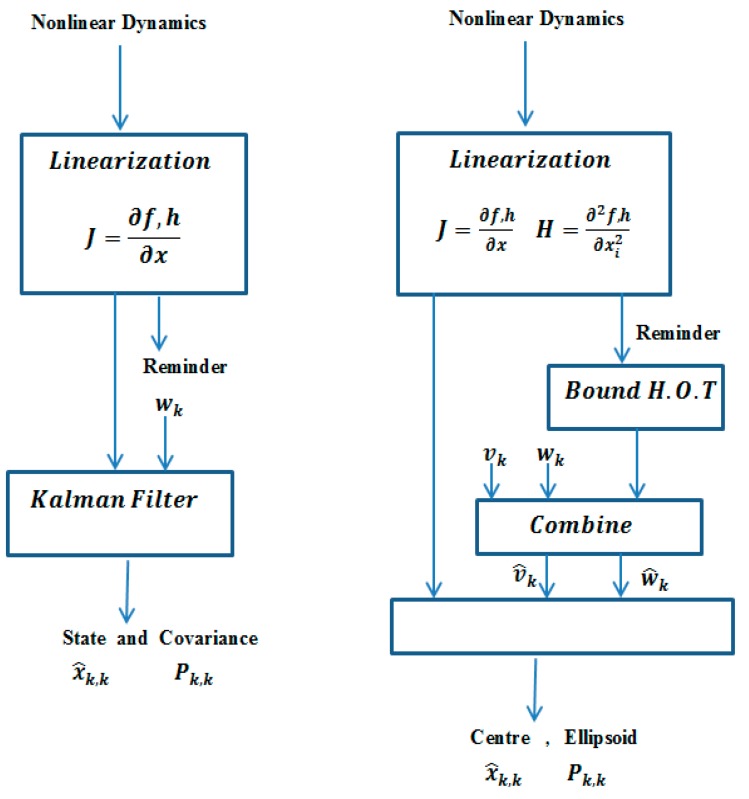
Simplified graphical representation of the extended Kalman filter (EKF) (left) and the nonlinear set membership filter (right) at each time step.

The approach here is to cast the nonlinear dynamics in a way that is suitable for implementation within the linear set-member filter framework. Specifically, the nonlinear dynamics are linearized about the current estimate in a manner that is similar to the EKF. The remaining terms are then bounded, and they are incorporated into the algorithm as additions to the process or sensor noise bounds [39]. This allows the solution to be guaranteed for nonlinear systems so long as the bound on the nonlinear term is guaranteed. The proposed method is compatible with many current robust control methods that require hard bounds, as well as with planning algorithms that require guaranteed uncertainty information. It is also computationally efficient for online recursive implementation. The new algorithm is termed the extended set membership filter (ESMF) [11]. Figure 2 shows the how ESMF works. 

Consider a general discrete non-linear system described as:

(15) $$x_{k+1}=f\left(x_{k},\;\;u_{k}\right)+w_{k}$$

with its nonlinear output:

(16) $$y_{k}=h\left(x_{k},\;\;u_{k}\right)+v_{k}$$

where $x_{i}$ ∈ $R^{n}$ is the system state and $y_{i}$ ∈ $R^{m}$ is the measurement output, f(.) and h(.) are both general nonlinear $C^{2}$ functions and the initial state $x_{0}$ is known to be bounded by an ellipsoid given as:

$$x_{0}\in E\left({\hat{x}}_{0},P_{0,0}^{-1}\right)⟺\left[x_{0}-{\hat{x}}_{0}\right){]}^{T}P_{0,0}^{-1}\left[x_{0}-{\hat{x}}_{0}\right]\leq 1$$

where $x_{0}$ is the center of the ellipsoid.

$w_{k}$ ∈ $R^{n}$ and $v_{k}$ ∈ $R^{m}$ are process and measurement noise, respectively, which are bounded at each time step *k* and satisfy the following inequalities:

$$w_{k}\in E\left(0_{nx1},Q_{k}\right)\;⟺\;w{\;}_{k}^{T}\;Q_{K}^{-1}w_{k}\leq 1\;\;\forall \;k\;v_{k}\in E\left(0_{mx1},R_{k+1}\right)\;⟺\;v{\;}_{k}^{T}\;R_{K}^{-1}v_{k}\leq 1\;\;\;\forall \;k$$

where $P_{k,K},Q_{k}$ and $R_{k}$ are symmetric and positive definite matrices.

At time step *k*, the goal is to characterize a set of states represented by a minimized ellipsoid that are consistent with the available measurements and a priori bound constraints; the true state is guaranteed to be contained in a resultant compact ellipsoid $E\left({\hat{x}}_{k},P_{k}\right)$.

Note that no assumptions on the structure of the noise are made except the bounds; hence, many types of uncertainties are included within this framework including Gaussian and non-Gaussian uncertainties.

Assuming that f(.) and h(.) are continuously differentiable, and for all estimated values ${\hat{x}}_{k-1}\;or\;{\hat{x}}_{k}$, Equation (15) is linearized around the current state ${\hat{x}}_{k}$ at time step k using Taylor expansion yields:

(17) $$x_{k+1}=f\left(x_{k}\right)|{\;}_{x_{k}={\hat{x}}_{k}}+\frac{df\left(x_{k}\right)}{dx}|{\;}_{x_{k}={\hat{x}}_{k}}\left(x_{k}-{\hat{x}}_{k}\right)+\cdots +O\left(x_{K}^{2}\right)+w_{k}$$

The extended set membership filter (ESMF) method considers the higher order terms (H.O.T. $O\left(x_{K}^{2}\right)$ or the remainder which is ignored in the traditional EKF) as a part of the process noise, and thus the linearized Equation (17) can be rewritten as:

(18) $$x_{k+1}=f\left(x_{k-1}\right)|{\;}_{x_{k-1}={\hat{x}}_{k-1}}+\frac{df\left(x_{k-1}\right)}{dx}|{\;}_{x_{k}={\hat{x}}_{k}}\left(x_{k-1}-{\hat{x}}_{k-1}\right)+\cdots \;\;\;\;\;\;\;\;+\frac{f{\left(x_{k}\right)}^{\left(n\right)}}{n!}|{\;}_{x_{k}={\hat{x}}_{k}}{\left(x_{k}-{\hat{x}}_{k}\right)}^{n}+R_{n}\left(x_{k}-{\hat{x}}_{k},X_{k}\right)+w_{k-1}$$

where $R_{n}\;\mathrm{is}$ a Lagrange remainder is term and $f{\left(x_{k}\right)}^{\left(n\right)}$ is the nth derivative. The term $X_{k}$ can take on any value over an interval for which $\left(x_{k}-{\hat{x}}_{k}\right)$ is defined. Thus, $R_{n}\left(x_{k}-{\hat{x}}_{k},X_{k}\right)$ can be bounded by simply defining the interval $X_{k}$ and evaluating $R_{n}\left(x_{k}-{\hat{x}}_{k},X_{k}\right)$ using interval mathematics. Using interval analysis, the Lagrange remainder term can be expressed as:

(19) $$R_{n}\left(x_{k}-{\hat{x}}_{k},X_{k}\right)=\frac{f^{\left(n+1\right)}\left({\bar{X}}_{k}\right)}{n!}{\left(x_{k}-{\hat{x}}_{k}\right)}^{n+1}$$

where the ${\bar{X}}_{k}$ is the state interval bound in which $\left(x_{k}-{\hat{x}}_{k}\right)$ is defined:

$${\bar{X}}_{k,\mp }^{i}={\hat{x}}_{k}^{i}\mp \sqrt{P_{k}^{i,i}\;\;}i=1,2,3\ldots ,n,$$

For a one-state (i.e., *n* = 1) linearization case with first order approximation (i.e., *nr =* 1), the state function in (9).

**Figure 3:**
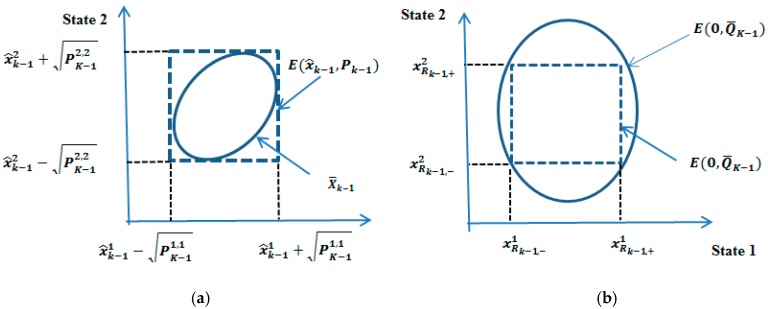
(**a**) Derivation of the interval X_k from P_((K,k)); (**b**) derivation of the bound on the study (illustration of ellipsoidal bound of linearization error).

The reminder or H.O.T. can be bounded by several ways. One approach is to choose the process and sensor noise ellipsoids large enough to assure that they bound both the original noise and the H.O.T. The approach here is to bind the H.O.T. using interval mathematics at each time step. The interval bound can then be bounded using an ellipsoid and combined with the original process noise bound. This approach is more amenable to on-line implementation as compared to similar approaches. Compared to the EKF, the ESMF now bounds the linearization error. In addition, the only restriction on the ESMF is that the Hessian and Jacobin must be continuous over the set of states. The procedure for bounding the general remainder $R_{n}\left(x_{k}-{\hat{x}}_{k},X_{k}\right)$ is shown pictorially in Figure 3 for a two state case. 

It is now possible to recursively estimate state ellipsoid sets of any nonlinear system for which the Jacobian and the Hessian are well defined.

An adaptive ellipsoidal SLAM algorithm can be summarized as follows (Algorithm 1):
**Algorithm 1. Adaptive Ellipsoidal SLAM**StartInitialization -${\hat{x}}_{1,1}=0$  $P_{k,k}$ = 0Get Observation – $k_{z}=1$        $z_{1}$ = get_observations; Looking for NAO markerswhile not_stopPrediction Step – (4)            Check for safe distance to move by sonar. Move command                No safe distance. Turn 180 degree[${\hat{x}}_{K+1},P_{k+1,k}$] = prediction (${\hat{x}}_{k/k},P_{k,k},u_{k})$$\beta_{Q_{k}}$ = $\frac{\sqrt{\mathrm{Tr}\left(Q_{k}\right)}}{\sqrt{\mathrm{Tr}\left({\bar{Q}}_{k}\right)}+\sqrt{\mathrm{Tr}\left(Q_{k}\right)}}$              ${\hat{Q}}_{k}=\frac{{\bar{Q}}_{k}}{1-\beta_{Q_{k}}}+\frac{Q_{k}}{\beta_{Q_{k}}},\beta_{Q_{k}}\in \left(0,1\right)$
$\beta_{Q_{k}}$ = $\frac{\sqrt{\mathrm{Tr}\left({\hat{Q}}_{k}\right)}}{\sqrt{\mathrm{Tr}\left(\emptyset_{k}P_{k}\emptyset_{k}^{T}\right)}+\sqrt{\mathrm{Tr}\left({\hat{Q}}_{k}\right)}}$PK+1,k=∅kPk1−βk∅kT+Q^kβk,βk ∈ (0,1)              ∅k=∂f(xk)∂xk| xk=x^kGet Observation (5)- Looking for NAO markers by turning head by 15 degrees.              If NAO find NAO markers to robot frame             Data Association (zk,x^k/k,Pk+1,k) Correction _Step - (${\hat{x}}_{k/k},P_{k+1,k},z_{k})$wk=Hk+1Pk+1,k1−ρkHk+1T+R^k+1ρk  $\rho_{k}$ ∈ (0,1)              $k_{k+1}=\frac{P_{k+1,k}}{1-\rho_{k}}H_{k+1}^{T}{\;w}_{k}^{-1}$
${\hat{x}}_{k+1}={\hat{x}}_{k+1,k}$ + kk+1[yk+1−h(x^k+1)]
           ${\bar{P}}_{k+1,k}=\frac{P_{k+1,k}}{1-\rho_{k}}-\frac{P_{k+1,k}}{1-\rho_{k}}{\;H}_{k+1}^{T}{\;w}_{k}^{-1}H_{k+1}\frac{P_{k+1,k}}{1-\rho_{k}}$
$\rho_{k}$ = $\frac{\sqrt{\mathrm{Tr}\left({\hat{R}}_{k+1}\right)}}{\sqrt{\mathrm{Tr}\left(H_{k+1}P_{k+1}H_{k+1}^{T}\right)}+\sqrt{\mathrm{Tr}\left({\hat{R}}_{k+1}\right)}}$Map_Step - (${\hat{x}}_{k+1/k+i},P_{k+1,k+1},z_{k})$ Add new Naomarks to the map        No Naomark –Turn Nao by 180 degree and go to step(5)$k_{z}=k_{z}+1$Check if iteration numbers are achieved   No Go to step (4)K = K + 1End
where $\rho_{k}\;\mathrm{and}\;\beta_{k}$ are filter parameters to be chosen online to minimize the ellipsoid. 

The obtained EKF/ellipsoidal SLAMs results are still not very satisfactory, presumably due to the range measurement bias and some other facts of imperfection like false positives in the data association and landmark description, etc. In SLAM, the state vector $s_{t}$ contains only landmark positions ($m_{x,i,}m_{y,i,}$) and robot poses $x_{t}.$ The SLAM alone is not expected to convey more information about landmarks details. The map representation does not include any information about landmark description in the environment and this will lead to false positives in the data association.

Accurate data association and landmark identification is very important in order to avoid convergence towards the wrong SLAM solution. Also, the computational cost of the EKF SLAM is quadratic with respect to a number of landmarks, i.e., *O* (*N*). It is a result of maintaining a full covariance matrix of state estimate. Also, the correction step of the EKF will touch every single element of the covariance matrix with a computation cost of *O* (*N*), which makes the problem intractable for cases with hundreds of landmarks.

To improve the EKF/ellipsoidal SLAM’s performance, including mapping in terms of reducing the computational effort, landmark identification and recognition, to simplify the data association problem and to improve the trajectory control of the algorithm, the robotic augmented reality (RAR) technique is introduced in this project and used to get better EKF/ellipsoidal SLAMs performance. An overview of augmented reality is included in the next section. Augmented reality is a recent technology which enables users to obtain additional preloaded information from the observation of a particular object.

### 3.3. Robotic Augmented Reality (RAR)

Researchers in many areas have been inspired by augmented reality (AR) in many areas including the medical, military, and entertainment fields. However, only a few applications are founded in robotics research, especially in the robotic navigation area. In this work, augmented reality technology has been integrated with EKF/ellipsoidal SLAM algorithms to improve their performances in real time. A review of RAR is introduced in the next section.

**Figure 4:**
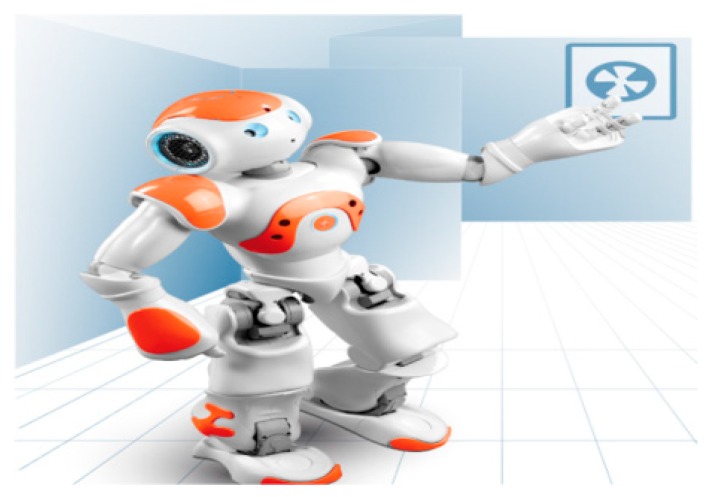
A talking objects participant places an augmented reality (AR) marker on a display case.

The fundamental idea of augmented reality (AR) is to mix the view of the real environment with virtual or additional computer-generated graphic content to improve our perception of the surroundings. An example of AR application for mobile devices to obtain information in the environment is shown in Figure 4.

Augmented reality is a subset of the more general area of mixed reality (MR) [40], which refers to a multi-axis spectrum of areas that cover virtual reality (VR), telepresence, augmented reality (AR) and other related technologies [41].

There are two main classes of augmented reality: marker-based and marker-less. In a marker-based augmented reality application, the images to be recognized and provided beforehand, which significantly simplifies the process of image recognition. Adding a few small, easy to understand bits of information to a real scene can help guide the robot to easily perform many tasks.

Augmented reality can be used to improve the development of robot and robot applications, such as robot navigation and human and robot interactions (HRIs). More and more studies have been conducted on AR for humanoid robots and some of the applications of AR to humanoid robots have been in path planning [42,43], where AR is used for drawing guide paths to provide a simple and intuitive method for interactively directing the navigation of a humanoid robot through complex terrain.

**Figure 5:**
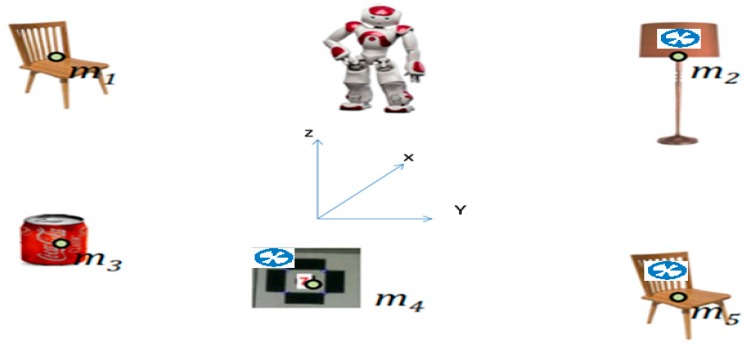
Augmented NAO mark in the environment.

The objective of augmented reality technology is to enhance the information acquisition of practical world by augmenting with computer-generated sensory inputs. We have integrated augmented reality with the EKF/ellipsoidal SLAM algorithms to improve SLAM performance and accuracy. SLAM performance is improved by presenting preloaded information to the robot through different AR markers placed in the robot’s indoor environment, and this information will help in the mapping process, simplifying the data association problem and reducing computational costs. Figure 5 demonstrates the outline of the navigation strategy. The same AR navigation module will be used as a part of a simultaneous localization and mapping system, which is developed for the same humanoid robot platform [44].

The SLAM new state vector can be represented by the following column vector including the AR marker IDs:

(20) $$s_{t}=\left(p_{x,t,}p_{y,t,}p_{\theta ,t,}\;ID_{1}\left(m_{x,1,}m_{y,1,}\right),ID_{2}(m_{x,2,}m_{y,2}\right)\ldots \ldots ID_{N}(m_{x,N,}m_{y,N}){)}^{T}$$

This new state vector will contain more information about the landmark IDs, which solves the landmark identification problem and simplifies the data association problem, because the corresponding landmark can be easily matched once the marked ID is re-observed, without having to compute the Mahalanobis distance frequently. This will optimize path planning and will reduce computational costs.

#### 3.3.1. Robotic Augmented Reality Implementation on the NAO Robot

The augmented reality and landmark-based SLAM approach is used for humanoid (NAO) robot applications in indoor environments.

In order to explain the details of the proposed algorithm, we start this section with a description of the NAO humanoid robot and how the NAO marks are employed. This background information is required before introducing distance estimation for NAO that is essentially provides a monocular vision system. We will then explain the NAO odometry problem and go through the derivation of the SLAM for this system. 

##### Platform and Software (NAO Humanoid Robot) 

NAO is a small humanoid robot, developed by the French company Aldebaran, and is designed to be versatile and affordable for educational research at universities, research centers, and also is a good candidate for social robots in applications such as museums, dance performances, etc. [45].

**Figure 6:**
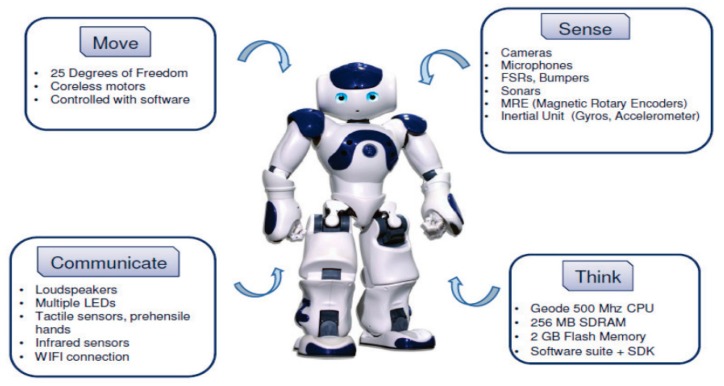
NAO robot capabilities and sensors.

The main version of NAO is the H25, which has 25 degrees of freedom. Nao is 58 cm height and about 5 kg weight. The robot also has an Intel ATOM Z530 1.6 GHz processor, with 1 Gb of RAM, as well as an Ethernet port, a Wi-Fi connection and a USB port [45] provides a summary of the NAO robot and its main features including movement, sensing, communication and acting. Gouaillier [46] describes the details of the mechatronic design of the NAO. Figure 6 shows the NAO robot’s main sensors.

In order to perceive its environment, Nao is equipped with several sensors, such as two cameras, four microphones, two sonars, two bumpers, and an accelerometer, to name a few. The two video cameras are located on its head; one is in the robot’s forehead (top camera) to view straight ahead of the robot, and the other one (lower camera) is located at its mouth to view the ground in front of the robot. In the NAO H25, which is the model used for our project, video cameras provide up to 1280 × 960 resolutions at 30 frames per second (fps). Cameras are crucial sensors for autonomous navigation

**Figure 7:**
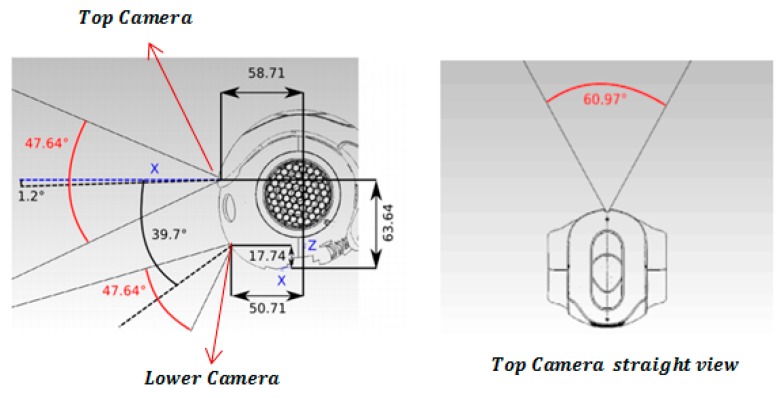
NAO cameras and their field of view.

The NAO cameras and their field of view and their positioning are described in Figure 7. 

It can be seen that there is no considerable overlap between the two video cameras’ fields of view. Therefore, the cameras do not provide stereo vision. This work utilized the NAO straight ahead camera as a monocular vision system (top camera) for perception of the world.

Each camera is an Aptina MT9M114, maximum resolution 1280 × 960 with an associated frequency of 5 Hz, and maximum frequency 30 Hz from 640 × 480 Video Graphics Array (VGA) (resolutions [45]. The resolution of the image is between QQVGA 80 × 60 and 4VGA 1820 × 960. The focal length of the camera is fixed, and its aperture angle is 60.9° horizontally and 47.6° vertically.

The acquisition system of the camera is a rolling shutter, that is to say the image is acquired line by line. This acquisition mode involves vertical deformation, which effects the image when the robot moves; the speed of movement of the head is greater than the acquisition speed of the columns. Settings such as white balance, brightness, or exposure time are adjustable. In practice, when the robot is moving, the images are subject to deformation because of strong kinetics and because the head is not stabilized except when the robot has both feet on the ground or is not making a step. 

NAO has a stable walking algorithm, described in [45]. A simple control system maintains its equilibrium (notably using the inertial unit and the Magnetic Rotary Encoder (MRE )sensors), but has no feedback on the actual movement of the robot. The control is thus in an open loop on the position.

On the software side, the NAO robot is equipped with the NAOqi operating system by the Softbank Robotics Community. The NAOqi OS [45] is the operating system of the robot and is an embedded GNU/Linux distribution based on Gentoo Linux. It was developed by Aldebaran to specifically fit the needs of the robot by providing and running programs and libraries that together transform it into a social robot. The main software of the NAOqi OS is NAOqi, which is responsible for running behaviors on the robot but can also be used to test code on a simulated robot on a PC. The NAOqi framework currently supports five programming languages: C++, Python, URBI, Java and Matlab. It has also been tested in the Microsoft.Net framework for the C#, F# and Visual Basic programming languages. Amongst the specified programming languages, Python and C++ are the most developed for the NAO, with extensive libraries. This work was mainly developed in the Python language using the Windows platform. The implementation methods were typical software engineering approaches, which included planning, developing, debugging and testing.

##### NAO Markers

The NAO’s vision recognition engine can be used is to recognize different objects. Some of the vision built-in functions and modules provided by Aldebaran are used, such as redBallDetection, to recognize balls and faceDetection to recognize faces. ALLandMarkDetection is a vision module in which the robot recognizes special landmarks with specific patterns on them. We refer to those landmarks as “NAO marks”. One can use the ALLandMarkDetection module for various applications. For example, they can be placed at different locations in the robot’s field of action. Depending on which landmark the robot detects, the information about the robot’s location can be inferred. Coupled with other sensory information, it is possible to build a rather robust localization module. This information will help with the estimation of the camera pose in the NAO space and the camera pose in the world space. Let us first discuss how to coordinate NAO marks in the robot’s camera [45].

**Figure 8:**
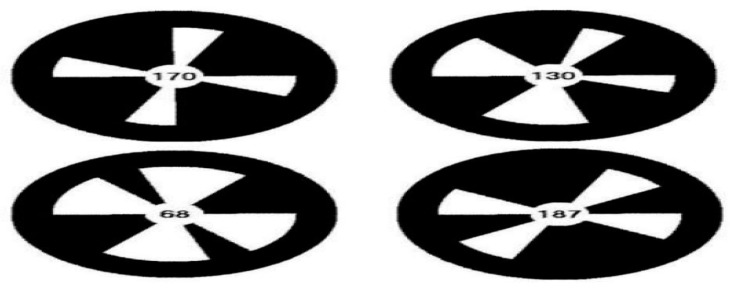
Some of the NAO markers.

Aldebaran robotics provides a total of 29 markers. Each of these markers has a unique shape that distinguishes it from the others. As shown in Figure 8, a NAO marker is a black circle with a white pattern in it. Encoded in the shape of the white pattern is the unique identity of the marker. When the NAO camera detects the NAO mark, it acquires useful information from these markers as we will see in next section [14]. 

As stated in the Aldebaran documents [45], the vision recognition module offers a simple approach to perceive the environment which is simple but presents several challenges. The first one is that the total number of NAO marks is limited, so if an application requires more of them, they need to be decoded again. The second is that these NAO marks require sufficient illumination, as correct decoding depends on contrast differences in the image. Proper illumination must be in the range of 100 lux and 500 lux. Lighting conditions below this range often result in the misidentification of the marker, or no detection at all in some cases. The third is that the tilt of the marker’s plane relative to the camera must be between +/− 60 degrees for detectability. For optimal performance, the NAO mark must also be in the direct line of sight of the robot. The final limitation is the size of the marker within the image and the range of detection by the camera. The minimum size is approximately 0.035 rad which corresponds to 14 pixels in a Quarter Video Graphics Array (QVGA) image, while the maximum size is approximately 0.40 rad or 160 pixels within the QVGA image. At this marker image size ranges and marker real size being 108.54 mm, the distance range for detection is from 30 cm to about 200 cm [45]. 

The NAO mark can correctly detect if the image has only 60˚ of inclination in relation to the camera. On the other hand, an advantage of the NAO mark is that its rotation has no influence on detection.

Also, it should be stated that the NAO can also recognize Quick Response (QR) codes; a QR code is a quick response code which is like a bar code in matrix form. It can store a lot of useful information. The NAO can be implemented to detect the QR code sign; the robot can then move closer to read it and finally work out its meaning. The AR markers (or NAO markers) do not have to be placed in any particular location as the NAO is programmed to recognize them and find where they are as it walks in its environment and then localizes them with respect to its pose as well as the global position.

**Figure 9:**
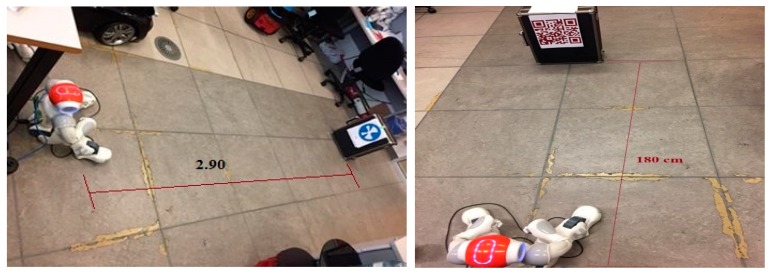
Detected NAO mark and QR codes by the NAO and their distances.

Our real-time experiments show that QR codes have more or less the same issues as the NAO markers. Firstly, the image resolution must be set according to the distance between the camera and the code itself. Secondly, the maximum distance for the NAO to be able detect the QR code is about 2 m, and this distance decreases when the QR code is not in straight view of the camera. Whereas our experiments show that NAO marks are very good to when used as landmark and produce better performance. By improving the NAO mark size and color as shown in figure below, the NAO is able to detect it up to 3.0–3.4 m from any camera angle. Figure 9 shows the detected NAO mark and QR codes by the NAO and their distances. 

The coordinates of the marker acquired by the robot’s camera are computed using the information shape of the marker. First, we need to know the physical dimension of the NAO mark detected. 

**Figure 10:**
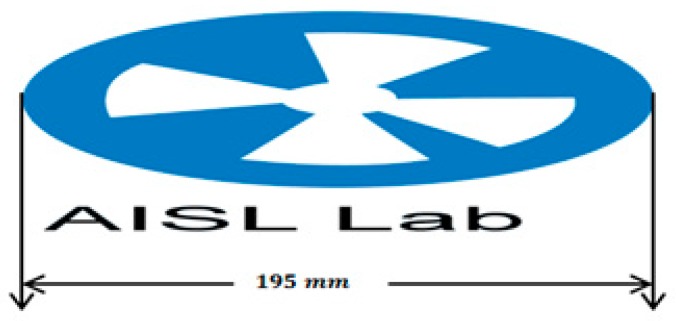
NAO mark dimension.

The marker size of the printed marker used in our study shown in Figure 10 is 195 mm with some information augmented to it. With this, we can calculate the distance of the marker from the robot, as explained bellow.

**Figure 11:**
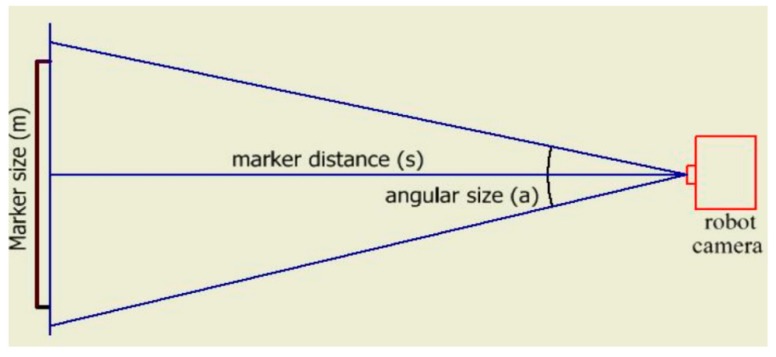
NAO camera angles.

In Figure 11, the variable (S) is the distance of the marker from the camera can be calculated using the angular size (a) and the marker size (m) as show in the following equation. The parameters sizeX and sizeY, for which identical values are given in the image of detected markers, are the dimensions from the center-most to the edge on the horizontal and vertical image axes, respectively.

(21) $$S=\frac{m/2}{\mathrm{atan}\left(a/2\right)}$$

In Equation (21), the angles alpha and beta are used to obtain the transformation from the robot to the landmark frame. To obtain the coordinate of the marker in the robot frame, a transformation from the camera frame to the robot frame is required. This transformation is performed using methods defined in the transform class of the implementation. The computation for this transformation is presented in Equation (22):

(22) $$\begin{array}{l} RobotToLandmark\\ \;\;\;\;\;\;=landmarkToCameraRotationalTransform\\ \;\;\;\;\;\;*\;landmarkToCameraTranslationalTransform*cameraToRobot \end{array}$$

The result is a transformation matrix which includes the (x, y, z) coordinates of the landmark in the robot frame. 

**Figure 12:**
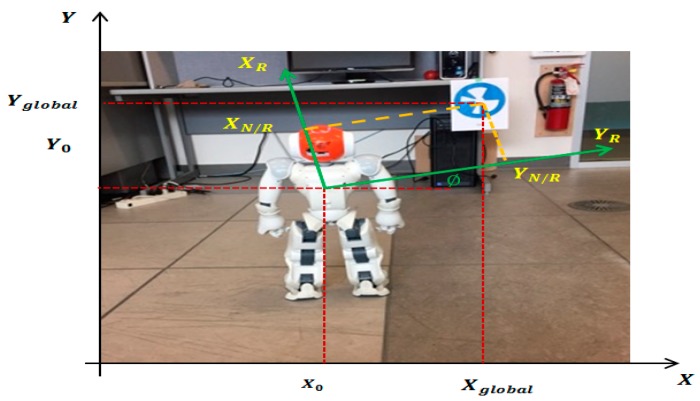
NAO mark global coordinates.

Figure 12 explains the transformation between the global frame of the world and local frames of the NAO robot and landmark (NAO mark).

In general, a 2D transformation between two frames is a combination of rotation and translation, written as:

(23) $$\left[\begin{array}{c} X_{global}\\ Y_{global} \end{array}\right]=\left[\begin{array}{cc} cos\emptyset & -\sin\emptyset \\ sin\emptyset & cos\emptyset \end{array}\right]\left[\begin{array}{c} X_{Naomark/robot}\\ Y_{Naomark/robot} \end{array}\right]+\left[\begin{array}{c} X_{0}\\ Y_{0} \end{array}\right]$$

where “robot” indicates marker coordinates in the robot frame ($X_{Naomark/robot},Y_{Naomark/robot}$) and $X_{0}$ and $Y_{0}$ are the robot’s location in the global frame. The angle ∅ denotes the orientation of the robot in the global frame. The equation can be rewritten as:

(24) $$\left[\begin{array}{c} \begin{array}{c} X_{global}\\ Y_{global} \end{array}\\ 1 \end{array}\right]=\left[\begin{array}{ccc} cos\emptyset & -sin\emptyset & X_{0}\\ sin\emptyset & cos\emptyset & Y_{0}\\ 0 & 0 & 1 \end{array}\right]\left[\begin{array}{c} \begin{array}{c} X_{Naomark/robot}\\ Y_{Naomark/robot} \end{array}\\ 1 \end{array}\right]$$

Now it’s possible to get the global coordinates of any NAOmark in the environment.

##### NAO Robot Odometry Problem

Bipedal walking robots present specific challenges depending on the environment in which they operate; they rarely achieve the desired trajectory because of the deviation generated during walking [47]. This problem is due to different circumstances such as robot manufacturing, wear and tear of mechanic parts, or variations in floor flatness, and due to partial hardware failures, out-of-spec components, the friction forces that are generated during motion, etc. [14].

**Figure 13:**
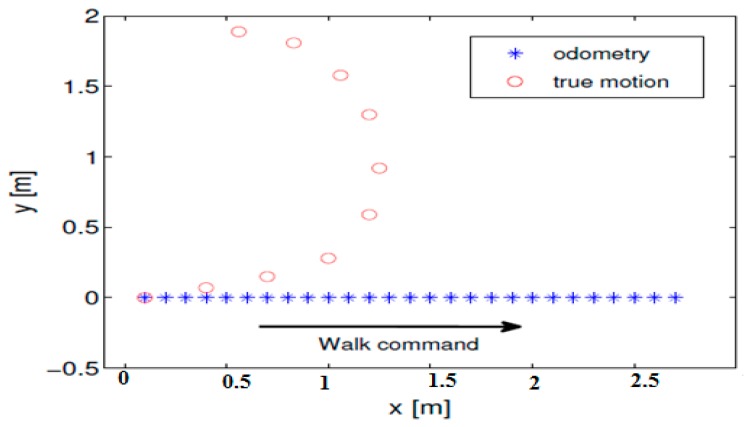
Actual robot trace and reported odometry for a straight walk command.

The odometry provided by the NAO robot firmware is computed from the robot model, i.e., step length and walk angle only, with no data from the inertial unit used (although it is available on the robot). The robot pose outputs from odometry are relative to some starting point and so only relative transformations to the previous poses are used in our localization system. The produced poses are known to be biased with an additive error, which is not negligible for legged robots. It was noted in the preliminary experiments that the odometry alone cannot be used for reliable robot navigation, as the resulting robot pose deviates greatly from the ground truth. Figure 13 shows the discrepancy between the actual robot trace and the reported odometry for a straight walk command. According to the odometry, the robot moved across a straight line because of the given command, whereas in reality the robot slipped to the left [48]. 

The NAO’s issue of deviation during walking will lead to errors in robot position and orientation after a few steps. Experiments show that the NAO’s orientation has a large error compared to its position error and as a result, all landmarks (NAO mark location) will have an inaccurate position value and consequently lead to a completely inaccurate map. Perception sensors which provide information relative to the environment (e.g., the camera) also have uncertainty in their measurements. Due to this high uncertainty in motion and perception, the estimation of the robot’s position, orientation and building of an accurate map can be improved using some filtering methods like the Kalman filter and its various non-linear extensions such as the extended Kalman filter (EKF) or the ellipsoidal SLAM as the goal of this paper.

The augmented reality and vision-based probabilistic landmark-based SLAM approach are implemented for humanoid (NAO) robot applications in indoor environments. It should be noted that the NAO’s mono-camera system is used to capture NAO marks as landmarks for SLAM applications as this humanoid robot do not have stereo vision, and due to its rather closed architecture, it is not straightforward to interface any external hardware (such as a stereo camera) to this platform. Also, adding additional hardware affects the walking stability of NAO. Finally, our solution alleviates the additional computational costs of image processing. By extension, an additional sensor (sonar) is used to find the safe distance for the NAO to move ahead and to avoid obstacles to perform the SLAM algorithm. Also, the sonar sensor can be used to find a safe distance and to perform SLAM faster. In the first stage, the application involves the NAO’s visual recognition ability to recognize some of the NAO markers and obtain some preloaded augmented information. The information can also later be used for the simplification of the data association problem in EKF/ellipsoidal SLAMs and help navigate in a dense environment with multiple landmarks and obstacles. In the next stage, the same AR module is transplanted into a vision-based autonomous humanoid robot to determine the position with respect to its environment. Markers whose locations are mapped based on the estimated location of the robot will be accurately updated in the update step of the EKF/ellipsoidal SLAMs. 

**Figure 14:**
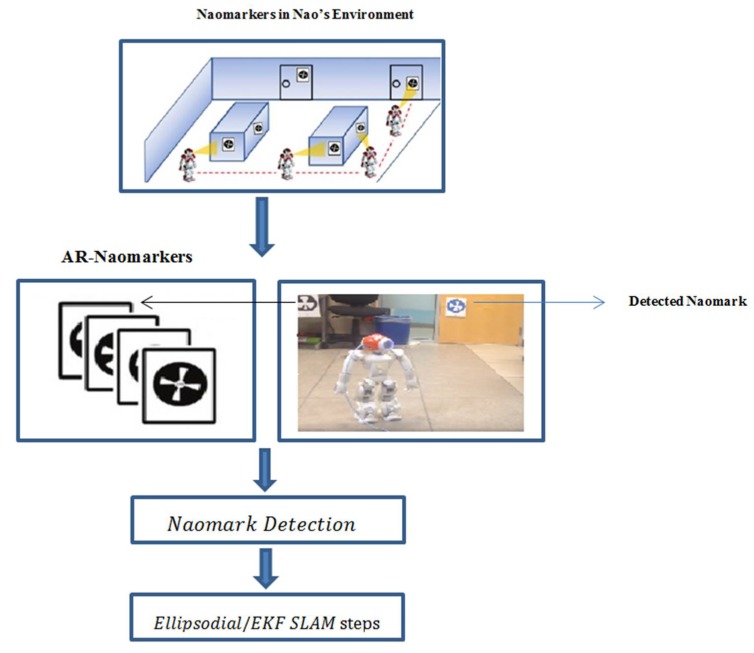
Augmented EKF/ellipsoidal SLAM outline strategy.

Initially, the EKF/ellipsoidal SLAMs perform regular map initialization processes and then start its regular motion function. However, before the EKF/ellipsoidal SLAMs conduct the observation function using the camera sensor, the NAO calls the landmark detection module aiming to find if there are NAO marks in the camera’s current field of view. This process is shown in Figure 14 and the algorithm below illustrates the process. The NAO starts turning its head by 15 degree increments and at the same time looking for NAO markers. In each head turn, and once the vision system detects any NAO marks, it will get the NAO mark’s data and all information associated with it and at the same time calculate the marker’s coordinates with respect to the robot’s frame. The designed system recognizes the location of a NAO marker from the image sequence taken from the environment using the NAO’s camera and adds the location information to the user’s view in the form of 2D objects and other information content using augmented reality (AR). 

The pseudo-code for the augmented EKF/ellipsoidal SLAM of the whole project with augmented reality is presented below (Algorithm 2).


**Algorithm 2. Augmented EKF/ellipsoidal SLAM**
StartInitialization - EKF/Ellipsoidal -SLAM Initialization, NAO Robot Initialization Get Observation –           Looking for NAO markers Yes - turning NAO’s head by 15 degrees.                      No - NAO markers Turn Nao by 180 degreewhile not_stopPrediction Step - Check safe distance to move by sonar. Move command                No safe distance. Turn Nao by180 degreeGet Observation (5) - Looking for NAO markers by turning head by 15 degrees.                  If NAO find NAO markers to robot frame                     Yes – Are/Is there any Augmented NAO markers                      Yes- go to step(6)                    No –Turn NAO by 180 degree and go to step(5) Data Association(6)-                   NAO markers matching and data-association simplification Correction _Step - Run standard EKF/ Ellipsoidal - SLAM update step.Augmented _Map Add new NAOmarkers to the mapCheck if iteration numbers are achieved      No Go to step 4End

NAO looks for the augmented NAO marker in its environment and the landmark detection module should report the detected NAO marker by circling them, with the mark’s ID displayed next to it. Once the right NAO marker is detected and the mark ID is retrieved, extra information can be achieved corresponding to the mark ID number, which is inspired by augmented reality. The sonar checks available space before the robot moves to the next prediction step. 

As we can see from the algorithm, the NAO starts turning its head by 15 degree steps and gets the NAO marker’s data at each step and at the same time calculates the marker’s coordinates with respect to the robot’s frame. Using augmented reality algorithms, whether the robot sees normal NAO markers or augmented NAO markers, the robot tries to find its location, simplify the data association and then goes to the update step. If the robot did not see any augmented NAO markers, it will go to the update step with the location calculated in the prediction step and run a regular EKF/ellipsoidal SLAM. All NAO markers are then mapped to the global location using EKF/ellipsoidal SLAM.

The integration of augmented reality with the EKF/ellipsoidal SLAM algorithms succeeded in the NAO robot based on the use of NAO marker recognition functions, as seen previously. Generally, the fundamental structures of EKF SLAM and ellipsoidal SLAM remain, while the augmented reality processes take place when the NAO marker is detected, where additional information is retrieved regarding the detected NAO marker. The main contribution of integrating augmented reality into both EKF SLAM and ellipsoidal SLAM is the use of this additional information to assist the robot in navigating tasks within a practical environment. 

The integration of augmented reality (AR) with EKF/ ellipsoidal SLAM is proposed and implemented on a humanoid robot in this paper. The goal has been to improve the performance of these SLAMs in terms of reducing the computational effort, simplifying the data association problem and to improving the SLAM algorithm. 

## 4. Experimental Results 

**Figure 15:**
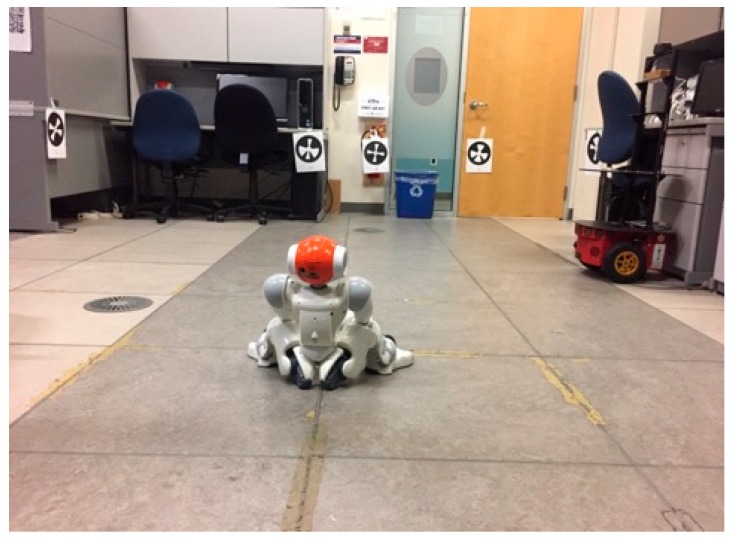
Experimental environment.

In this section, we evaluate the effectiveness of the ellipsoidal SLAM through simulations and experiments in real-time. We compared ellipsoidal SLAM with EKF SLAM. The experiment results conducted in the Autonomous and Intelligent Systems Laboratory (AISL) as shown in Figure 15. The NAO markers are placed on different unknown locations in the lab, as seen in the Figure 15, at the same height as the of NAO. The robot stands up and starts looking for landmarks by turning its head by 15 degrees. 

First the robot is placed at pose [0,0,0] and then a straight move command is applied (moveTo(0.25,0,0)) on the NAO on the iteration step to move to (4, 0, 0). The NAO starts to deviate to the left side after the few first steps. With this error, the odometry alone cannot be used for reliable navigation as explained in Section NAO Robot Odometry Problem. The goal is to leave the NAO moving without any control and to find its location and map its environment.

**Figure 16:**
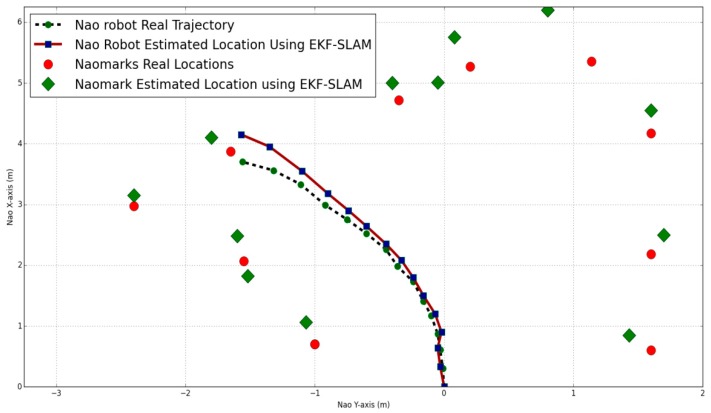
NAO location and NAO markers positions with real-time implementation of EKF SLAM.

Figure 16 shows the robot’s trajectory and the NAO marker locations estimated by EKF SLAM under the Gaussian noise assumption. The figure shows that the robot’s position started at the origin. It’s clear that the EKF is able to acceptably estimate the robot’s location compared to the real robot location with inaccurate orientation. We noticed from the experiments that the uncertainty of the robot’s motion after passing the first two landmarks increased. The EKF SLAM algorithm minimized this error again when the system observed new landmarks but the estimation errors of the EKF SLAM increased under Gaussian noise. 

The NAO marker locations were subject to more errors compared to the real location. This was due to the robot’s orientation error. The NAO V4 provides poor orientation values from the Inertial Measurement Unit (IMU) which will lead to false NAO marker locations and the data association problem.

In practice, the NAO robot detected some falsely-positive NAO markers. The false positive problem happened firstly because of illumination, as mentioned previously, and because of the NAO camera’s field of view; the NAO detects only one NAO marker at once so if two NAO markers are located in the same field of view of the robot camera, the image processing algorithm for NAO marker recognition fuses them to generate a new NAO marker shape and consequently the result will be a false positive NAO markER. Figure 16 shows some false positive NAO markers detected by the NAO camera.

These false positive NAO markers cause inaccurate localization and mapping processes. 

**Figure 17:**
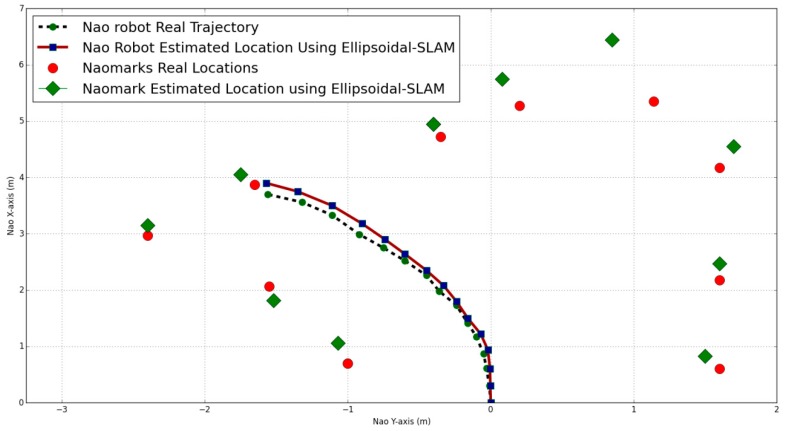
NAO location and NAO marker positions using ellipsoidal SLAM.

Figure 17 shows the robot trajectory and the NAO marker locations estimated by ellipsoidal SLAM. 

In ellipsoidal SLAM, the noise is bounded using the ellipsoidal method. It is clear that in some parts, ellipsoidal SLAM performance is similar to the standard EKF SLAM, but the ellipsoidal SLAM bounds the estimation errors after every iteration.

The implemented standard that the EKF/ellipsoidal SLAMs still suffer from are false positives in data association, computational costs and the SLAM vector does not include any information about landmark description in the environment. Furthermore, the standard ellipsoidal SLAM required a trial and error process to select its parameters and this process was more tedious in real time towards robust SLAM. 

### Augmented EKF/Ellipsoidal SLAM Results

**Figure 18:**
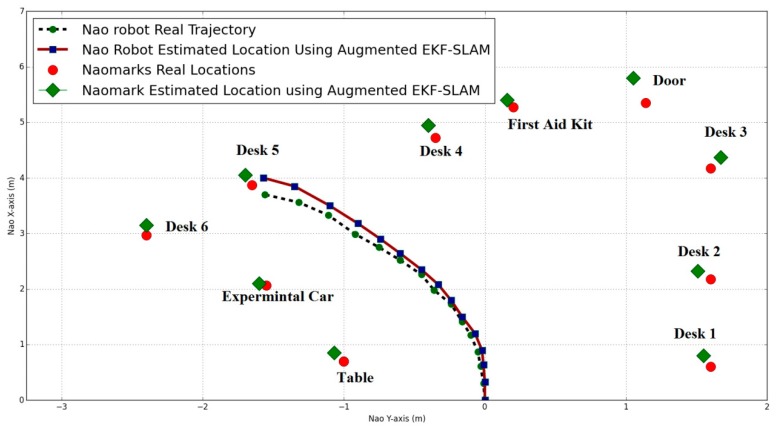
NAO location and NAO marker positions using augmented EKF SLAM.

In this part, we employed the augmented reality fundamentals to improve the mapping term in EKF/ellipsoidal SLAM. Figure 18 shows the estimated NAO robot pose in each iteration step and NAO marker positions using augmented EKF SLAM. AR solves the false positive NAO markers and provides more information about the landmarks in the environment. It improves the SLAM algorithm by providing what type of object is in the environment.

**Figure 19:**
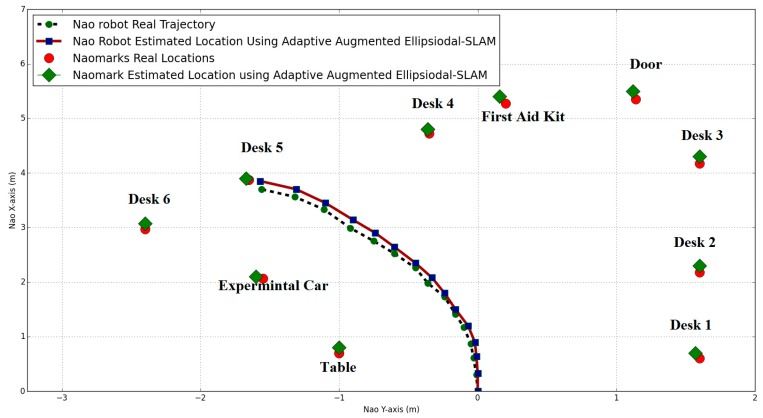
NAO location and NAO marker positions using adaptive augmented ellipsoidal SLAM.

In Figure 19, the NAO markers are placed in the robot’s path. While the robot moves between these landmarks, the estimated robot position is minimized when the robot detects any augmented NAO marker on its route. 

The new SLAM state vector now has more information about the object in its environment with its locations as follows.

$$s_{t}=\left(p_{x,t,}\;p_{y,t,}\;p_{\theta ,t,}\;Table\left(m_{x,1,}\;m_{y,1,}\right),A\;Car(m_{x,2,}\;m_{y,2}\right)\ldots \;\ldots ,Table(m_{x,N,}\;m_{y,N}){)}^{T}$$

This will improve the SLAM algorithm and this will easily solve the data association problem because the corresponding landmark can be easily matched once the marker ID is re-observed, thus reducing the computational cost.

It was noted that the uncertainty of the robot’s motion increased when the robot was running under the standard EKF SLAM (no augmented NAO marker had been detected) and the EKF SLAM process could not minimize the error. However, when the system was augmented by new NAO markers, it corrected its position and minimized the error. Furthermore, the estimated NAO marker positions were improved by using augmented reality. Additionally, the robot’s position in the augmented ellipsoidal SLAM was better than in the augmented EKF SLAM. These observations support our conclusion that generally, the ellipsoidal SLAM algorithm improves the motion model and decreases its error.

In ellipsoidal SLAM, the parameters which minimize the ellipse are calculated by trial and error and adaptive augmented ellipsoidal SLAM finds these parameters online in every iteration to improve the accuracy of the SLAM algorithm. Table 1 illustrates these values.

We employed the root mean square (RMS) method for the computational cost in the comparison of the accuracy and consistency of the implemented SLAMs. The RMS is defined as:

(25) $$e=\sqrt{\frac{\sum_{i=1}^{n_{x}}{\left(x_{i}-{\hat{x}}_{i}\right)}^{2}}{n_{x}}}$$

where $x_{i}$ is the truth value for the state vector, while ${\hat{x}}_{i}$ is the estimated state value. Furthermore, the estimated positions of landmarks were also evaluated. Some detailed results calculated by the RMS are listed in Table 2. They are the average values over real-time experiments. As the table shows, the NAO’s IMU has the largest error in the y-axis, with a $45^{{}^{\circ}}$ orientation error after the final step of movement. EKF SLAM reduces the heading error by 66.6% of the total error of the IMU, but this is not enough to perform an accurate SLAM. Adaptive augmented EKF SLAM reduced this error by 88%. Thus, we can draw the conclusion that adaptive augmented EKF SLAM is much more effective for improving accuracy.

**Figure 20:**
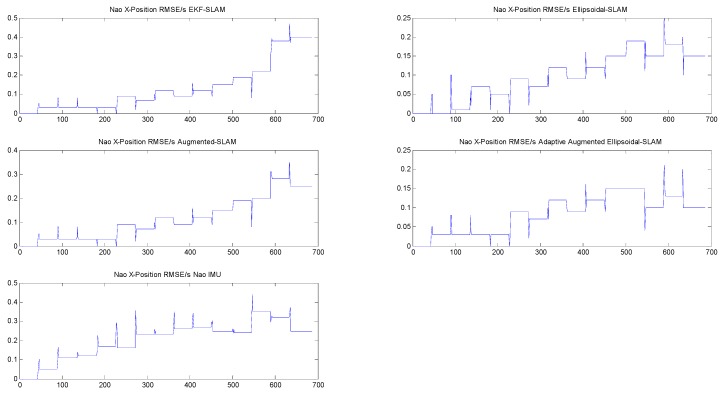
The root mean square (RMS) errors of NAO x-positions of the implemented SLAM.

**Figure 21:**
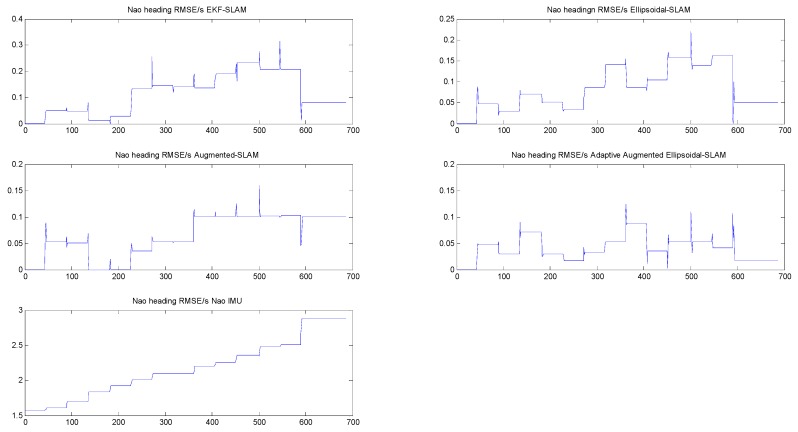
The RMS errors of the NAO heading of the implemented SLAM.

Figure 20 and Figure 21 compare the RMS errors of the NAO’s *x*-position and the headings of the implemented SLAM. Obviously, the trend of errors in adaptive augmented Ellipsoidal SLAM is steady and smaller than that of EKF/ellipsoidal SLAM and augmented EKF SLAM, including both the NAO position errors and heading errors. All of the errors decrease by about 88 percent under the adaptive augmented ellipsoidal SLAM. The steady RMSE in the figures means that the NAO is not moving in that specific time duration and performing the observation step, looking for NAO markers in the environment. 

**Figure 22:**
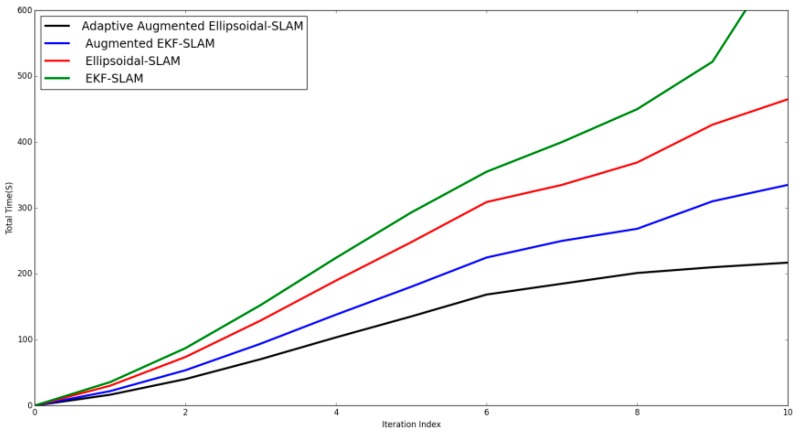
Steady-state time elapsed for the implemented SLAM algorithms.

Moreover, the time to reach steady-state estimation should also be taken into account. Figure 22 shows the total time required for the filters to reach steady-state estimation. For adaptive augmented ellipsoidal SLAM, there is a slightly difference compared to augmented EKF SLAM. The adaptive augmented ellipsoidal SLAM still has a speed advantage over EKF and ellipsoidal SLAM, while preserving a more accurate and consistent estimate. Thus, we can see that adaptive augmented ellipsoidal SLAM is clearly time-saving compared to EKF and ellipsoidal SLAMs. Adaptive augmented ellipsoidal SLAM needs 120s after the 6th step to provide stable, accurate, and consistent estimates.

The results of the experiments performed shows that the standard EKF/ellipsoidal SLAM algorithms have very similar performance. Noticeably, Adaptive augmented ellipsoidal SLAM SLAM algorithm improves the localization and mapping process with very small error. 

## 5. Conclusions

The ultimate goal of this project was to study various SLAM algorithms using monocular vision as the only information source to model the environment and provide accurate solutions and implement them on the NAO humanoid robot. Augmented EKF/ellipsoidal SLAM solutions, which integrate augmented reality techniques with ellipsoidal SLAM were explained and tested on the NAO humanoid robot to enable the NAO move through its environment. The fundamentals of each step of mapping and localization have been explained and implemented with the NAO robot for EKF/ellipsoidal SLAM algorithms. The results of the experiments performed in the AISL lab showed that regular EKF/ellipsoidal SLAM algorithms have very similar performance in terms of mapping and in most simulated cases, ellipsoidal SLAM was more robust in modeling the motion errors. Furthermore, errors are bounded and modeled by ellipsoid sets without assuming a Gaussian environment, so we have more realistic error modeling capable of building maps for both indoor and outdoor environments. The augmented EKF/ellipsoidal SLAM systems give more accurate robot pose estimations and NAO marker positions. Noticeably, adaptive augmented ellipsoidal SLAM improves localization and mapping. Besides the improvement of the algorithm itself to include some landmark information, we have reported great improvement in the consistency and accuracy. Adaptive augmented ellipsoidal SLAM results decreased IMU errors by 88%, while EKF/ellipsoidal SLAM reduced this in the range of 66%–75%. These results are good to be used with the NAO robot for indoor applications. The adaptive augmented ellipsoidal SLAM was able to determine the robot’s position each time with very small errors when compared to localization done by odometry or by regular EKF/ellipsoidal SLAM. The mapping process and data association are solved successfully despite having some false positive NAO markers using RAR. 

The experiments show that the robot can find its location and build an acceptable map around itself successfully at each step in an acceptable time using adaptive augmented ellipsoidal SLAM. 

## Figures and Tables

**Table 1:** Ellipse parameters for adaptive augmented ellipsoidal SLAM.

	Estimated Parameters of Adaptive Augmented Ellipsoidal SLAM														
$\beta_{k}$	1	2	3	4	5	6	7	8	9	10	11	12	13	14	15
	0.21	0.19	0.18	0.16	0.174	0.17	0.158	0.148	0.16	0.19	0.17	0.20	0.18	0.16	0.14
$\rho_{k}$ × $10^{-5}$	49	47	45.5	46	54	44.5	43	41	40.4	40	43	46	43	41	39

**Table 2:** Comparisons of the performances of implemented SLAM algorithms.

Algorithm	Nao x-Pose Error/m	Nao y-Pose Error/m	Nao Heading Error/rad	Naomarks-x Error/m	Naomarks-y Error/m
NAO IMU	0.7120	48.9912	0.78558	-	-
EKF SLAM	0.0184	0.18451	0.17975	0.1413	0.4022
Ellipsoidal SLAM	0.0180	0.12016	0.12128	0.1173	0.4143
Augmented EKF SLAM	0.0178	0.1439	0.077072	0. 5901	0.2153
Adaptive Augmented Ellipsoidal SLAM	0.0163	0.1001	0.034711	0.2566	0.1024

## Abbreviations

The following abbreviations are used in this manuscript:

SLAM	Simultaneous Localization and Mapping
AEKF	Augmented Extended Kalman Filter
EKF	Extended Kalman Filter
RAR	Robotic Augmented Reality
CML	Concurrent Mapping and Localization
ESMF	Extended Set-Membership Filter
